# Efficient Indium-Mediated Dehalogenation of Aromatics in Ionic Liquid Media

**DOI:** 10.3390/molecules18010398

**Published:** 2012-12-28

**Authors:** Álvaro F. Cañete, Cristian O. Salas, Flavia C. Zacconi

**Affiliations:** Facultad de Química, Pontificia Universidad Católica de Chile, Vicuña Mackenna 4860, Macul 78204336, Región Metropolitana, Santiago, Chile

**Keywords:** indium, ionic liquids, dehalogenation, carbene complexes, reduction

## Abstract

An efficient indium-mediated dehalogenation reaction of haloaromatics and haloheteroaromatics in ionic liquids has been studied. This method is simple and effective in the presence of [bmim]Br. Furthermore, this methodology is environmentally friendly compared with conventional ones.

## 1. Introduction

In the field of synthetic organic chemistry, dehalogenation reactions are particularly interesting in processes that involve them as starting materials or as intermediates. Nevertheless, halogenated organic compounds also represent an important class of environmental pollutants. From this point of view, new dehalogenation methods are an important goal [1]. 

The ease with which dehalogenations proceed depends on the nature of the carbon-halogen bond, carbon atom hybridization, substituent effects, and the presence of diverse functional groups or halogens on the scaffold structure. In addition, it has been determined that the reductive dehalogenation of organic halides generally follows the reactivity order I > Br > Cl > F. Therefore, due to this difference, selective dehalogenations of compounds containing more than one halogen become possible [2,3,4]. Furthermore, it has been found that current methods are more efficient when the reaction is performed on iodine derivatives, while for chloro and bromo compounds these procedures have been less satisfactory.

Typical procedures have generally used electrochemical, photochemical, and ultrasonic techniques. Moreover, among the more common chemical hydrodehalogenation methods for organic halides, catalytic hydrogenations with metal compounds are widely used [2], although some of these are troublesome to execute [5,6,7,8,9]. Within the broad range of conditions for carrying out such reactions, the use of indium is of particular interest because it can be utilized in different organic solvents (THF, toluene, *etc.*), in water, and without solvent. 

Furthermore, another important advantage of indium metal is associated with its first ionization potential (In, 5.8 eV), as low as those of Li (5.4 eV) and Na (5.1 eV) [10], which promotes single electron transfer (SET) processes between the metal and aromatic compounds. Besides, this metal is stable in air and its toxicity is lower compared with that of other metals [11].

In spite of these interesting properties, the use of In to reduce carbon-halogen reactions has been relatively unexplored. In early attempts, Ranu *et al.*, developed a chemoselective reduction methodology for carbonyl compounds and α-halobenzylhalides using In(0) in water under sonication [11]. Later, Hirasawa *et al.*, achieved a series of dehalogenation reactions of iodoquinoline derivatives in aqueous media [12]. However, the very low solubility of some organic substrates in water is a serious limitation in terms of reactivity, which obliged us to explore these reactions in organic solvents such as THF, acetonitrile or ethanol with a consequent decrease in yields.

With the aim of contributing to the development of new synthetic methodology in the field of indium chemistry, and as a preliminary study, we propose the use of an ionic liquid as an alternative solvent to carry out dehalogenation reactions. Furthermore, this is also an attractive green chemistry approach as it does not require toxic solvents or harmful chemicals [13]. The results of this study could be useful to introduce an efficient method for the degradation of halogenated environmental pollutants.

## 2. Results and Discussion

Dehalogenation of aromatic compounds was investigated using a series of reactions under several conditions. At first, these reactions were tested in the presence of organic and aqueous solvents. Bromobenzene was used as a target molecule in this preliminary study. To this end, one equivalent of bromobenzene was treated with one equivalent of In(0) in water at room temperature or under reflux during 14 h, with complete recovery of the halo precursor in both cases (Table 1, entries 1 and 2). Another approach for the dehalogenation reaction used anhydrous THF or a THF/H_2_O (1:1) mixture (Table 1, entries 3–6), but the same results were obtained. After these failures, it was decided to use another reaction medium. As ionic liquids (IL) have well-known properties as green solvents, we tested the same procedure only changing the solvent medium [14,15]. With this modification, treatment of bromobenzene with [bmim]Cl (1-butyl-3-methylimidazolium chloride) and [bmim]Br (1-butyl-3-methylimidazolium bromide) afforded benzene in good to excellent yield (Table 1, entries 8–13). However, when other counterions and cations were used, e.g., [bmim]PF_6_ (1-butyl-3-methyl-imidazolium hexafluorophosphate), [bmim]BF_4_ (1-butyl-3-methylimidazolium tetrafluoroborate), [bmpy]F_3_CSO_3_ (1-butyl-1-methylpyrrolidinium trifluoromethanesulfonate) and TBAF (tetrabutyl-ammonium fluoride) the reactions did not proceed (Table 1, entries 7, 15–17).

In order to ensure the validity of the results shown above for bromobenzene, we proceeded to extend this method to chloro- and iodobenzene. When [bmim]Cl was used, moderate yields of the dehalogenated product were obtained (Table 1, entries 8–10), which is consistent with the lower reactivity of the carbon-halogen bond. In contrast, when halobenzenes **1** were treated with [bmim]Br under the same reaction conditions, dehalogenated products **2** were obtained quantitatively (Table 1, entries 11–13). 

**Table 1:** Optimization of the indium-mediated dehalogenation reaction ^a^.

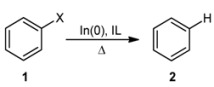
Entry	Solvent	X	Temperature (°C)	Yield ^b^ (%) 2	Recovery ^b^ (%) 1
1	H_2_O	Br	rt	-	>99
2	H_2_O	Br	100	-	>99
3	THF	Br	rt	-	>99
4	THF	Br	65	-	>99
5	THF/H_2_O	Br	rt	-	>99
6	THF/H_2_O	Br	65	-	>99
7	TBAF	Br	95	-	>99
8	[bmim]Cl	Br	95	70	30
9	[bmim]Cl	Cl	95	60	40
10	[bmim]Cl	I	95	3	97
11	[bmim]Br	Br	95	>99	-
12	[bmim]Br	Cl	95	>99	-
13	[bmim]Br	I	95	>99	-
14	[(*d_3_*)-bmim]Br	Br	95	>99 ^c^	-
15	[bmim]BF_4_	Br	95	-	>99
16	[bmim]PF_6_	Br	95	-	>99
17	[bmpy]F_3_CSO_3_	Br	95	-	>99

^a^ In (1 equiv.), in IL (2 equiv.), 14 h; ^b^ Values of **1** and **2** were determined by GC-MS; ^c^^2^H-benzene was obtained.

Our experimental results indicate that [bmim]Br is the most effective solvent for this reaction. In order to obtain some further insight into the dehalogenation reaction under these conditions, we investigated the behavior of other haloaromatic systems using indium in [bmim]Br. The yields of dehalogenated products were in the moderate to excellent range (Table 2). To evaluate the reactivity of the halogen atom, chloro-, bromo- and iodoaromatic compounds were used; lower yields were found for those substrates containing a chlorine atom as the leaving group (Table 2, entries 1 and 9) while for derivatives containing the other halogens yields were found to be nearly quantitative in all cases (Table 2, entries 6–8, and 10–12).

**Table 2:** Dehalogenation of haloaromatics compounds using indium metal in IL ^a^.

Entry	Substrate	Product		Yield ^b^ (%)	Recovery ^b^ (%)
1	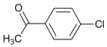	**3**	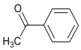	20	80
2	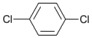	**4**	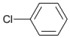	>99	-
3	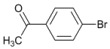	**3**	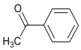	80	20
4	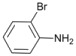	**5**	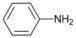	76	24
5	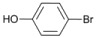	**6**	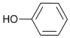	10	90
6	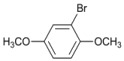	**7**	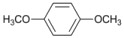	>99	-
7	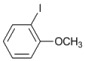	**8**	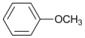	>99	-
8	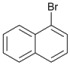	**9**	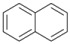	>99	-
9	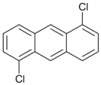	**10**	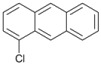	11	89
10 ^c^	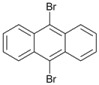	**11**	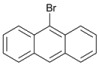	>99	-
11 ^c^	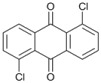	**12**	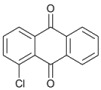	>99	-
12	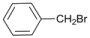	**13**	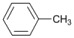	>99	-

^a^ In (1 equiv.), in IL (2 equiv.), 95 °C, 14 h; ^b^ Values of substrate and product were determined by GC-MS; ^c^ Reaction time: 5 h.

**Scheme 1:**
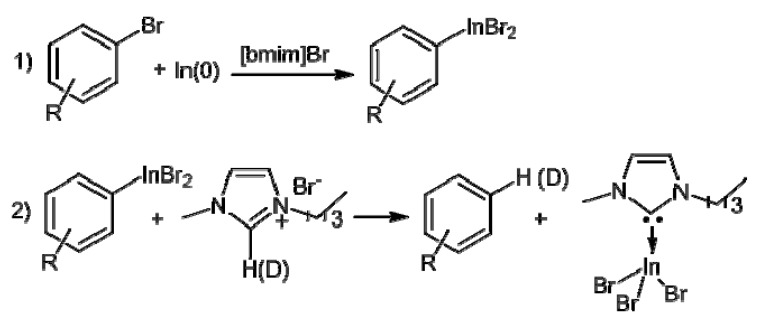
Proposed dehalogenation mechanism.

A preliminary study of the effects of other groups on the aromatic rings on the regioselectivity of this reaction was also done. In this investigation ester, nitro and nitrile groups were not considered to avoid side reactions [16,17]. It was found that the yield from *p*-bromophenol (Table 2, entry 5) diminished, a situation that agrees with the proposed mechanism due to the acidity of the phenol group (Scheme 1). In contrast, for halogenated acetophenones the yields depend on the intrinsic reactivity of the halogen (Table 2, entries 1 and 3). The same behavior was found when there are methoxy and amino groups on the aromatic system (Table 2, entries 4, 6, and 7). In the cases of dihalogenated substrates (Table 2, entries 9–11), the process was exclusively a monodehalogenation. We were encouraged to investigate the reactivity of benzyl bromide under the same reaction conditions, and found that the benzylic position was also dehalogenated in >99% yield (Table 2, entry 12). It is noteworthy that this reaction is not affected by the presence of electron-withdrawing or electron-donor groups on the substrate; side products were not observed in any case.

In order to extend this new dehalogenation methodology and gain deeper insight into its characteristics, we investigated the behavior of several heteroaromatic systems (Table 3). In the case of pyridine derivatives we found that the yields depend on the position, number and character of the halogen [18]. When a chlorine atom is at C-2, the reaction did not occur, while conversion was quantitative for the bromine derivative (Table 3, entries 1,2). However, when the bromine is at C-3, the reduction yield was moderate (Table 3, entry 3). Therefore, for pyridines with more than one halogen substituent, the reaction depends on their nature and location. In the case of 2-chloro-3-bromopyridine this process is regioselective for position 3 (Table 3, entry 4), whereas with 2-fluoro-3-chloropyridine (Table 3, entry 5) the reaction did not proceed. This is probably attributable to the stability of the C-F bond that makes it hard to cleave [16,17]. However, when an electron withdrawing group such as trifluoromethyl is *para* to fluorine (in the pyridine ring) the reaction can occur at more than one position, replacing the chlorine or fluorine atom due to weakening of the C-F bond (Table 3, entry 6). These results indicate that electronic effects in the dihalo-heteroaromatic ring can play a pivotal role in the selective dehalogenation process mediated by indium [19].

Finally, we evaluated the behavior of 2,5-dihalothiophenes (Table 3, entries 7 and 8). When 2,5-diiodothiophene was subjected to the standard conditions, the reaction proceeded quantitatively giving the monoiodo compound, and the monobromo analogue was similarly formed from dibromotiophene, confirming that the higher susceptibility of the halogen toward elimination is in accordance with its intrinsic properties as a leaving group.

With this last experiment, we can conclude that appropriate ionic liquid media allow the facile preparation of dehalogenated compounds. Therefore, it can be suggested that the IL behaves as a promoter aside from its role as a highly polar medium

**Table 3:** Dehalogenation of haloheteroaromatics compounds using indium metal in IL ^a^.

Entry	Substrate	Product		Yield ^b^ (%)	Recovery ^b^ (%)
1	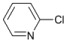	**14**	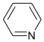	-	>99
2	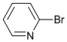	**14**	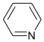	>99	-
3	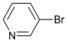	**14**	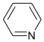	60	40
4	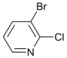	**15 ^c^** **16 ^d^**	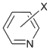	20 ^c^ 80 ^d^	-
5	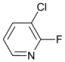	**17**	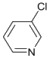	-	>99
6	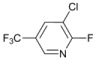	**18 ^e^** **19 ^f^**	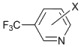	60 ^e^30 ^f^	10
7	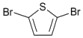	**20**	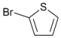	90	10
8	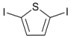	**21**	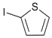	>99	-

^a^ In (1 equiv), in IL (2 equiv), 95 °C, 14 h; ^b^ Values of substrate and product were determined by GC-MS; ^c^ 3-bromopyridine; ^d^ 2-chloropyridine; ^e^ 2-fluoro-5-(trifluoro-methyl)pyridine; ^f^ 3-chloro-5-(trifluoromethyl)pyridine.

A plausible mechanism for the reductive dehalogenation of haloaryl compounds by In(0) in the [bmim]Br (Scheme 1) includes a single electron transfer from the metal surface to the aryl halide to form the dihalogenated indium salt (ArInBr_2_) [20]. Once this species is obtained, it abstracts a proton from a [bmim]Br molecule to generate a carbene intermediate, which constitutes a stable complex with InBr_3_, in agreement with previous investigations [21,22]. Moreover, the formation of the homo-coupling dimer did not occur, it was confirmed by GC-MS.

The higher efficiency of this reaction in [bmim]Br in comparison with [bmim]Cl can be explained invoking Pearson acid-base theory (HSAB). In this regard, the softness of the indium atom would determine the preference for Br^−^ (soft base) as the counterion instead of Cl^−^ (hard base) favoring the more stable ArInBr_2_ intermediate. To ascertain that the proton transfer is actually from IL, [(*d*_3_)-bmim]Br [23], was used as the IL, and the labelled aryl compound was obtained in excellent yield (Table 1, entry 14).

## 3. Experimental

### 3.1. General Methods

All starting materials were commercially available and used as received without further purification. TLC was performed on silica gel 60 F_254_, using aluminum plates and visualized with vanillin stain and UV lamp. Flash chromatography was carried out on handpacked columns of silica gel 60 (230–400 mesh). ^1^H-NMR (400 MHz) spectra were recorded at using CDCl_3_ as the solvent and TMS as internal standard (0.00 ppm). ^13^C-NMR (100 MHz) spectra were recorded with reference to the CDCl_3_ signal at 77.15 ppm. GC-MS experiments were made on a Clarus 680 gas chromatograph/SQ 8T mass spectrometer system (PerkinElmer), ionization by EI^+^ at 70 eV.

### 3.2. General Procedure for the Dehalogenation of Haloaromatics

A mixture of bromobenzene (47 mg, 32 μL, 0.30 mmol), indium powder (45 mg, 0.39 mmol) and [bmim]Br (132 mg, 0.60 mmol) was stirred for 14 h at 95 °C. Then, the resulting mixture was treated with water (2 mL), extracted with Et_2_O (3 × 2 mL), dried over anhydrous MgSO_4_ and evaporated under reduced pressure. The residue was analyzed by GC-MS and products characterized by ^1^H- and ^13^C-NMR. 

### 3.3. Characterization Data

*Benzene* (**2**). ^1^H-NMR: δ 7.83 (s, 6H). ^13^C-NMR: δ 129.19. GC-MS: *m/z* (%) = 51 (17), 78 (M^+^, 100).

*^2^H-Benzene* (**2**). ^1^H-NMR: δ 7.36 (s, 5H). ^13^C-NMR: δ 133.15, 129.03, 126.03, 124.98. GC-MS: *m/z* (%) = 77 (25), 78 (100), 79 (M^+^, 40).

*1-Phenylethanone* (**3**). ^1^H-NMR: δ 7.76 (d, *J *= 7.5 Hz, 2H), 7.31–7.37 (m, 1H), 7.20-7.26 (m, 2H), 2.35 (s, 3H). ^13^C-NMR: δ 197.23, 136.74, 132.46, 128.12, 127.83, 25.98. GC-MS: *m/z* (%) = 59 (100), 74 (79), 120 (M^+^, 7).

*Chlorobenzene* (**4**). ^1^H-NMR: δ 7.36–7.11 (m, 5H). ^13^C-NMR: δ 134.30, 129.80, 128.70, 126.51. GC-MS: *m/z* (%) = 51 (33), 78 (100), 113 (M^+^, 82), 115 (M^+^+2, 28).

*Phenylamine* (**5**). ^1^H-NMR: δ 7.35 (m, 2H), 6.89–7.03 (m, 1H), 6.81 (d, *J *= 8.0 Hz, 2H), 3.72 (s, 2H). ^13^C-NMR: δ 147.36, 129.37, 118.38, 115.10. GC-MS: *m/z* (%) = 59 (92), 74 (89), 93 (M^+^, 100).

*Phenol* (**6**). ^1^H-NMR: δ 7.19 (t, *J *= 7.2 Hz, 2H), 6.85-6.95 (m, 1H), 6.82 (d, *J *= 8.1 Hz, 2H), 6.15 (bs, 1H). ^13^C-NMR: δ 155.02, 129.84, 121.10, 115.36. GC-MS: *m/z* (%) = 66 (40), 94 (M^+^, 100).

*1,4-Dimethoxybenzene *(**7**). ^1^H-NMR: δ 6.83 (s, 4H), 3.76 (s, 6H). ^13^C-NMR: δ 153.76, 114.69, 55.73.GC-MS: *m/z* (%) = 95 (35), 123 (100), 138 (M^+^, 58).

*Methoxybenzene* (**8**). ^1^H-NMR: δ 7.23 (t, *J *= 7.2 Hz, 2H), 6.90 (t, *J *= 6.9 Hz, 1H), 6.87 (d, *J *= 6.9 Hz, 2H), 3.69, (s, 3H). ^13^C-NMR: δ 159.68, 129.46, 120.65, 113.94, 54.98. GC-MS: *m/z* (%) = 65 (95), 78 (94), 108 (M^+^, 100).

*Naphthalene* (**9**). ^1^H-NMR: δ 7.95–8.07 (m, 4H), 7.60-7.68 (m, 4H). ^13^C-NMR: δ 133.45, 127.89, 125.83. GC-MS: *m/z* (%) = 64 (52), 102 (50), 127 (M^+^−1, 100).

*1-Chloroanthracene *(**10**). ^1^H-NMR: δ 8.88 (s, 1H), 8.01 (s, 1H), 7.57–7.67 (m, 3H), 7.42 (d, *J *= 6.7 Hz, 1H), 7.20–7.30 (m, 3H). ^13^C-NMR: δ 134.72, 132.64, 128.47, 127.13, 126.49, 124.49, 122.93. GC-MS: *m/z* (%) = 88 (31), 178 (100), 213 (46), 247 (M^+^, 20), 249 (M^+^+2, 12).

*9-Bromoanthracene* (**11**). ^1^H-NMR: δ 8.88 (s, 1H), 8.46 (d, *J *= 7.2 Hz, 2H), 8.03 (d, *J *= 7.2 Hz, 2H), 7.78 (dd, *J *= 7.2 Hz, *J *= 8.1 Hz, 2H), 7.62 (dd, *J *= 7.2 Hz, *J *= 8.1 Hz, 2H). ^13^C-NMR: δ 133.91, 130.99, 129.71, 128.47, 126.13, 125.21, 122.93. GC-MS: *m/z* (%) = 88 (89), 176 (90), 256 (M^+^, 99), 258 (M^+^+2, 100).

*1-Chloroanthra-9,10-quinone* (**12**). ^1^H-NMR: δ 8.14 (d, *J *= 8.1 Hz, 2H), 7.92 (d, *J *= 8.1 Hz, 2H), 7.76 (m, 3H). ^13^C-NMR: δ 182.42, 181.79, 138.12, 135.56, 134.20, 131.34, 130.70, 129.34, 128.47, 127.43, 44.57, 45.21. GC-MS: *m/z* (%) = 186 (78), 214 (100), 241 (M^+^, 92, 243 (M^+^+2, 28).

*Toluene* (**13**). ^1^H-NMR: δ 7.55–7.61 (m, 5H), 2.75 (s, 3H). ^13^C-NMR: δ 137.83, 129.14, 128.29, 125.58, 21.18. GC-MS: *m/z* (%) = 65 (16), 91 (M^+^−1, 100), 92 (256 M^+^).

*Pyridine* (**14**). ^1^H-NMR: δ 8.31 (d, *J *= 5.9 Hz, 2H), 7.27–7.31 (m, 1H), 6.88–6.96 (m, 2H). ^13^C-NMR: δ 149.11, 134.98, 123.11. GC-MS: *m/z* (%) = 52 (89), 79 (M^+^, 100).

*3-Bromopyridine* (**15**). ^1^H-NMR: δ 8.66 (d, *J *= 7.6 Hz, 1H), 8.47 (s, 1H), 7.77 (d, *J *= 7.7 Hz, 1H), 7.14 (d, *J *= 7.6 Hz, 1H). ^13^C-NMR: δ 151.00, 147.80, 138.39, 124.55, 120.78. GC-MS: *m/z* (%) = 51 (33), 78 (100), 157 (M^+^, 28), 159 (M^+^+2, 30).

*2-Chloropyridine* (**16**). ^1^H-NMR: δ 8.44 (d, *J *= 8.4 Hz, 1H), 7.70 (dt, *J *= 7.7 Hz, *J *= 7.3 Hz, 1H), 7.39 (d, *J *= 7.4 Hz, 1H), 7.28 (dt, *J *= 7.3 Hz, *J *= 7.4 Hz, 1H). ^13^C-NMR: δ 151.39, 149.65, 138.64, 124.33, 122.18. GC-MS: *m/z* (%) = 51 (40), 78 (100), 113 (M^+^, 89), 115 (M^+^+2, 28).

*2-Fluoro-5-(trifluoromethyl)pyridine* (**18**). ^1^H-NMR: δ 8.21 (s, 1H), 7.98 (d, *J *= 5.8 Hz, 1H), 7.16 (d, *J *= 5.8 Hz, 1H). ^13^C-NMR: δ 162.03, 147.19, 139.08, 128.08, 124.77, 108.37. GC-MS: *m/z* (%) = 115 (52), 146 (78), 165 (M^+^, 100).

*3-Chloro-2-(trifluoromethyl)pyridine* (**19**). ^1^H-NMR: δ 8.41 (s, 2H), 7.90 (s, 1H). ^13^C-NMR: δ 150.09, 146.56, 137.48, 133.92, 125.73, 124.77. GC-MS: *m/z* (%) = 69 (38), 146 (42), 181 (M^+^, 100), 183 (M^+^+2, 24).

*2-Bromothiophene* (**20**). ^1^H-NMR: δ 7.14 (dd, *J *= 7.1 Hz, *J *= 7.0 Hz, 1H), 6.99 (dd, *J *= 7.0 Hz, *J *= 6.9 Hz, 1H), 6.80 (dd, *J *= 7.1 Hz, *J *= 6.9 Hz, 1H). ^13^C-NMR: δ 129.88, 127.68, 127.02, 112.21. GC-MS: *m/z* (%) = 57 (29), 83 (80), 162 (M^+^, 99), 164 (M^+^+2, 100).

*2-Iodothiophene* (**21**). ^1^H-NMR: δ 7.18 (dd, *J *= 7.1 Hz, *J *= 7.0 Hz, 1H), 7.03 (dd, *J *= 7.0 Hz, *J *= 6.9 Hz, 1H), 6.83 (dd, *J *= 7.1 Hz, *J *= 6.9 Hz, 1H). ^13^C-NMR: δ 130.22, 128.02, 127.36, 112.55. GC-MS: *m/z* (%) = 83 (52), 126 (16), 210 (M^+^, 100).

## 4. Conclusions

Dehalogenation of haloaromatics and haloheteroaromatics in an ionic liquid were successfully accomplished. Although the scope and limitations of this new method have not been fully established, it is a promising candidate for use as an alternative solution for the dehalogenation of aromatic or heteroaromatic intermediates. Besides, it constitutes an inexpensive method that avoids the use of other toxic and expensive reagents, such as Bu_3_SnH or NaBH_4_ [20,21]. Finally, it is an efficient new method for the preparation of regioselectively deuterated compounds.
